# Case report: Double adjacent ventral slot in two medium-sized breed dogs

**DOI:** 10.3389/fvets.2024.1346816

**Published:** 2024-05-15

**Authors:** Razvan Grigore Cojocaru, Bogdan Sicoe, Cristina Gaspar, Alexandra Grigoreanu, Gabriel Orghici, Ioan Tibru, Radu Lacatus

**Affiliations:** ^1^Faculty of Veterinary Medicine, University of Life Sciences “King Mihai I”, Timisoara, Romania; ^2^Faculty of Veterinary Medicine, University of Agricultural Sciences and Veterinary Medicine, Cluj-Napoca, Romania

**Keywords:** double adjacent ventral slot, cervical disk disease, medium-sized dogs, IVDE, tetraplegia

## Abstract

Two medium-sized, 7-year-old dogs, with no previous history of pain, presented with acute neurologic symptoms consistent with intervertebral disk disease. Both cases had CT, where cervical pathology was identified. In one dog, the diagnosis was singular extensive cervical disk herniation with possible epidural hemorrhage and in the other, the diagnosis was multiple-site cervical disk herniation. The first dog, a Shar-Pei, underwent treatment with two standard adjacent ventral slots between the C4–C5 and C5–C6 intervertebral disk spaces and a fenestration between the C3 and C4 intervertebral disk spaces. The second case, a beagle, underwent a double adjacent standard ventral slot between the C5–C6 and C6–C7 intervertebral disk spaces. Both dogs recovered uneventfully after the surgery and showed no signs of recurrence during a 2-year follow-up period. This is the first detailed report of the use of a double adjacent ventral slot as a treatment for spinal decompression in medium-sized dogs with multiple-site spinal cord compression.

## Introduction

Intervertebral disk disease (IVDD) is one of the most common causes of spinal cord dysfunction in dogs that present acute neurologic deficits (1).

Clinical signs can vary depending on the type of herniation, the location, and the extent of the compression (2).

The most common location for disk herniation in dogs is in the thoracolumbar region of the vertebral canal, with 16 to 25% of cases reported in the cervical region (3). The majority of dogs with cervical disk herniation present with a nucleus pulposus extrusion rather than an annulus protrusion (1, 3–7).

The cervical disk extrusions are most commonly encountered in chondrodystrophic dogs and they usually have an acute onset (1, 8).

Traditionally, disk herniations were divided into two distinct types: Hansen Type 1 (IVD extrusion) and Hansen Type 2 (IVD protrusion). A third type was also described as an acute non-compressive or high-velocity low-volume disk disease (9–12).

More recently, Fenn et al. (4) suggested the need for more precise classification, considering the latest advances in veterinary diagnostic techniques (magnetic resonance imaging), proposing the following specific pathologies individually: Hansen type 1/acute (IVD extrusion); Hansen type 2/chronic (IVD protrusion); acute IVD extrusion (HT1) with extensive hemorrhage; acute non-compressive nucleus pulposus extrusion (ANNPE); hydrated nucleus pulposus extrusion (HNPE); intradural/intramedullary IVD extrusion (IIVDE); traumatic IVD extrusion; and fibrocartilaginous embolic myelopathy (FCEM).

In the cervical region, the incidence rate of IVDE varies between 16 and 25% and is more commonly encountered in small breed dogs, where C3–C4 is the most commonly reported site. Hansen type 2 disk protrusion is more commonly found in large breed dogs between the C5 and C6 intervertebral disk spaces (3, 13).

Conservative treatment and surgical treatment have been described for canine patients with cervical disk extrusion, with the ventral approach to the cervical vertebrae being the most common choice of decompression treatment (14, 15).

The advantages of the ventral slot include minimal dissection to access the vertebral bodies to perform the slot and also a prophylactic fenestration to the adjacent disks. The main disadvantages reported for this technique include venous sinus hemorrhage, hypoventilation, respiratory distress, cardiac arrhythmias, instability/ subluxation of the vertebras, spinal cord trauma, and insufficient exposure to intraforaminal and lateral disk extrusion and postoperative infection (1, 16–18).

A double or even triple adjacent ventral slot has been described for multiple disk herniations or for lesions that have a longer extent but have not been reported in medium- and large-breed dogs (19, 20).

## Case description

Two medium-sized (10–25 kg) (21), middle-aged dogs, namely, a Shar-Pei (case 1 - entire female, 18 kgs, 7 years and 10 months) and a Beagle (case 2—entire male, 25 kgs, 7 years and 5 months) were referred for having an acute onset of neurologic signs (ataxia and neurologic deficits).

The owners reported an acute onset of the neurologic signs, which started 24–48 h before the presentation. At the time of presentation, both dogs did not have any history of other health problems and did not show any signs of pain before the onset of the clinical signs. A general and neurologic examination was conducted and nothing abnormal was detected apart from the following clinical signs:

Case 1 was non-ambulatory, tetraplegic, and had a moderate pain reaction on the hyperextension and hyperflexion of the neck. The withdrawal reflex and patellar reflex were reduced in the right pelvic limb and deep pain perception was reduced in both pelvic limbs.

Case 2 was non-ambulatory, tetraplegic with spontaneous severe pain reaction at any attempt of hyperflexion of the neck. Withdrawal reflexes were reduced in both pelvic limbs and deep pain perception.

These findings were consistent with upper motor neuron deficit in the thoracic limbs and lower motor neuron deficit in the pelvic limbs.

For both cases, a blood sample was taken from the jugular vein and submitted to the laboratory for biochemistry and hematology analysis, and no abnormality was detected.

Following the blood analysis, the dogs were submitted for a CT scan of the cervical region for diagnosis purpose. The CT scan was performed under sedation as described: a 20 G IV catheter was placed in the right cephalic vein and a 0.1 mg/kg butorphanol with 0.01 mg/kg of medetomidine was administered intravenously. The positioning of the dog was in dorsal recumbency. The CT findings were as follows:

### Case 1

**Figure 1:**
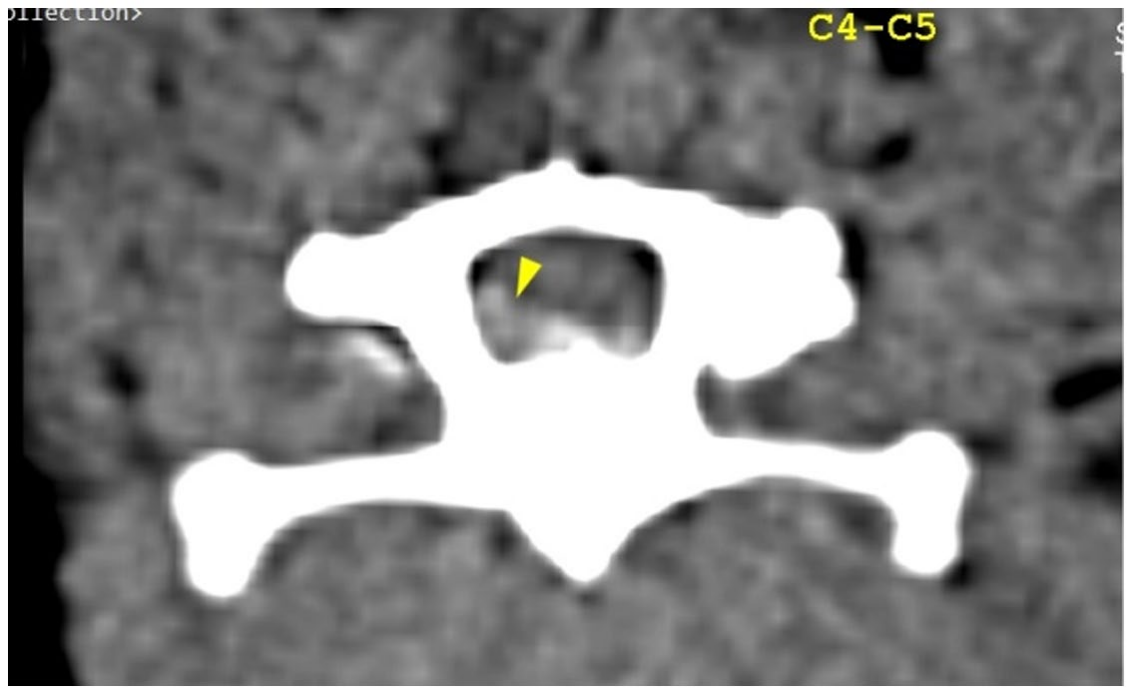
Transverse section of the C4–C5 intervertebral disk space. Yellow arrowhead highlights mild hyperattenuating extruded disk material and possible hemorrhage, located ventral and left lateral to the spinal cord.

**Figure 2:**
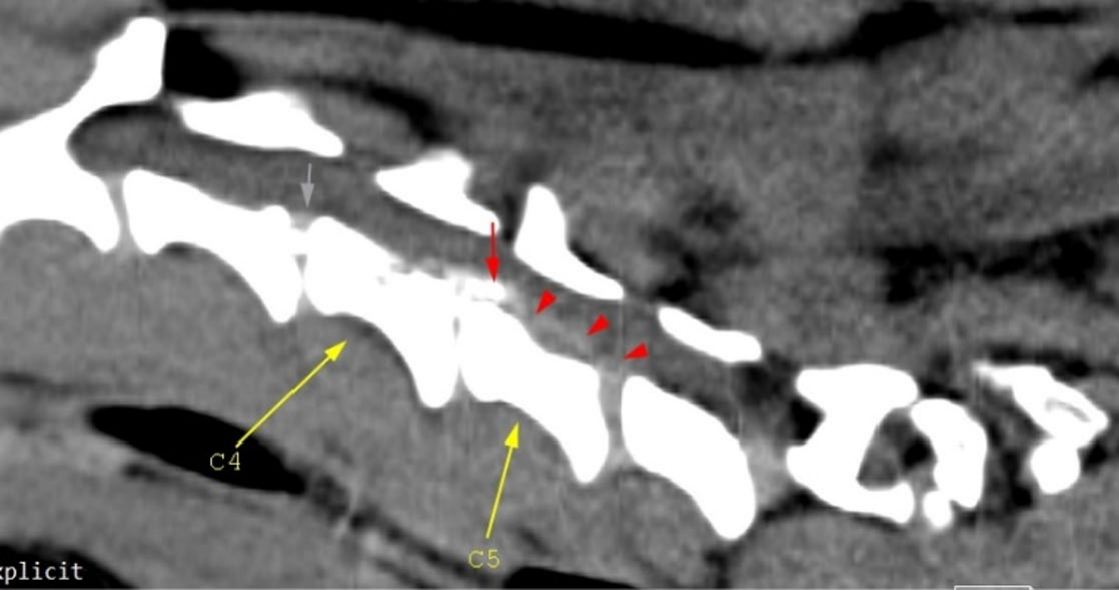
Mid-cervical sagittal view. The gray arrow depicts *in situ* C3–C4 disk mineralization; the red arrow highlights mineralized herniated disk material (C4–C5), and the red arrowheads highlight mild hyperattenuating disk material and possible hemorrhage. Malalignment of the cervical-thoracic segment is observed and this is due to the positioning of the dog.

**Figure 3:**
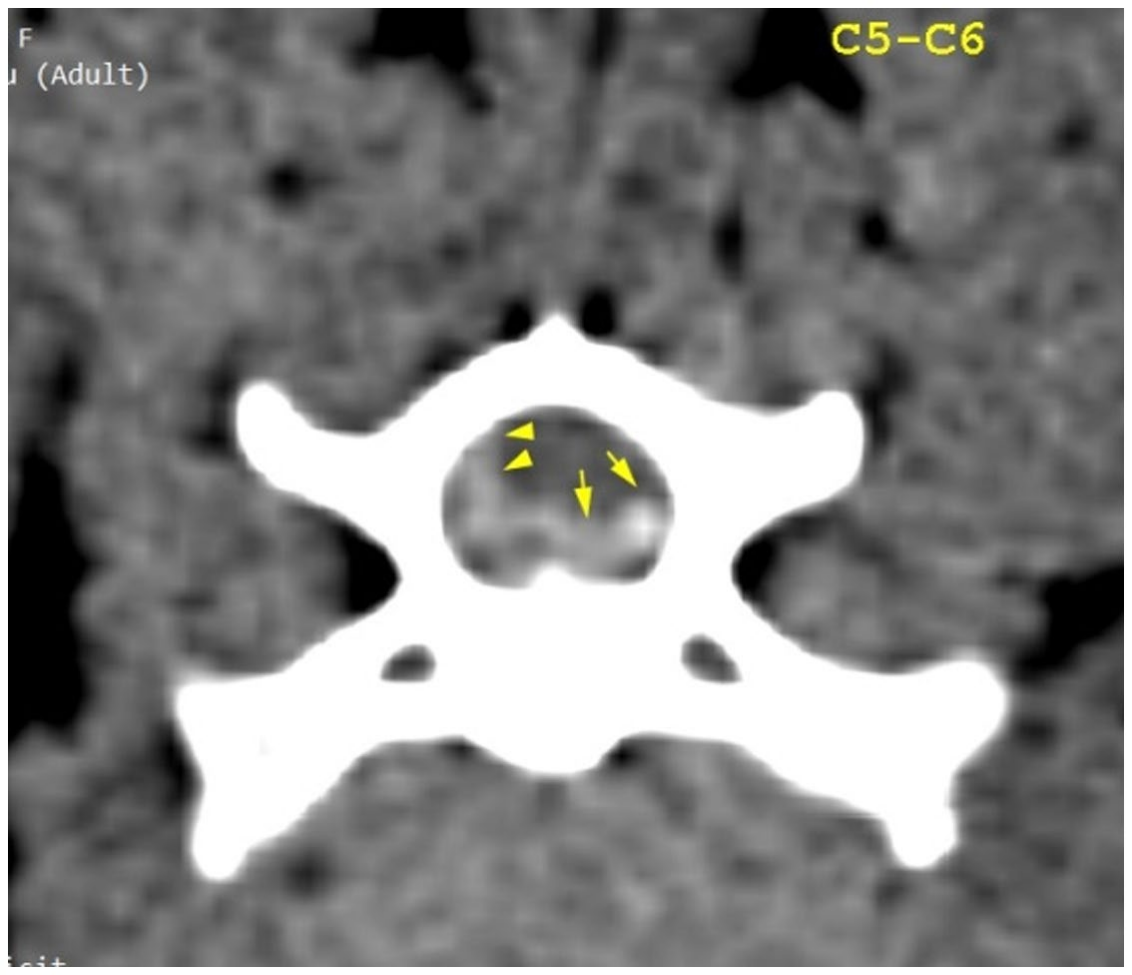
Transverse section of the C5–C6 intervertebral disk space. Yellow arrowheads highlight mild hyperattenuating herniated disk material and possible hemorrhage; yellow arrows highlight hyperattenuating disk material.

The CT scan showed hyperattenuating material present in the vertebral canal at the level of C4–C5, with the intervertebral space at this level being visibly narrowed. The material was located ventral and left lateral to the spinal cord. Caudally, the mild hyperattenuating material extended to the caudal part of the C5 vertebral body. The material presented higher Hounsfield unit values (88–101 HU) compared to the medulla (29–44 HU) and occupied 47.8% of the vertebral canal diameter (Figures 1, 2). Ventral extradural material that was isoattenuating to the spinal cord was present at the level of the C5–C6 disk (Figure 3). The C3–C4 intervertebral space was visibly narrowed, with *in situ* mineralized material and no visible spinal cord compression (Figure 2). These findings were consistent with C4–C5 disk extrusion with moderate to severe compression and C5–C6 mild Hansen type 1 extrusion.

### Case 2

**Figure 4:**
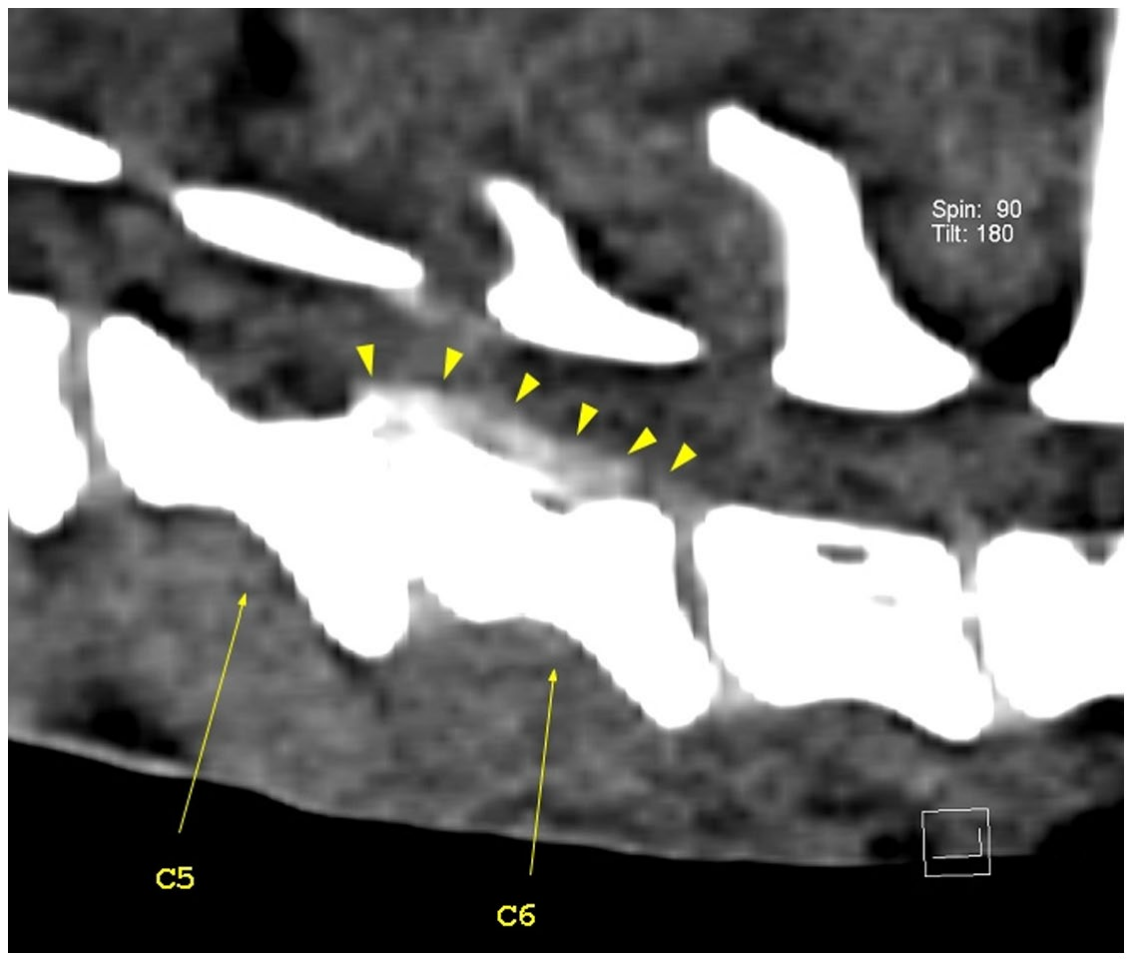
Sagittal view of the caudal cervical segment of the vertebral column. The C5–C6 intervertebral disk space is narrower than the adjacent ones, with visible heterogenous hyperattenuating herniated disk material (yellow arrowheads). The herniated disk material extends caudally to the caudal part of the C7 vertebral body.

**Figure 5:**
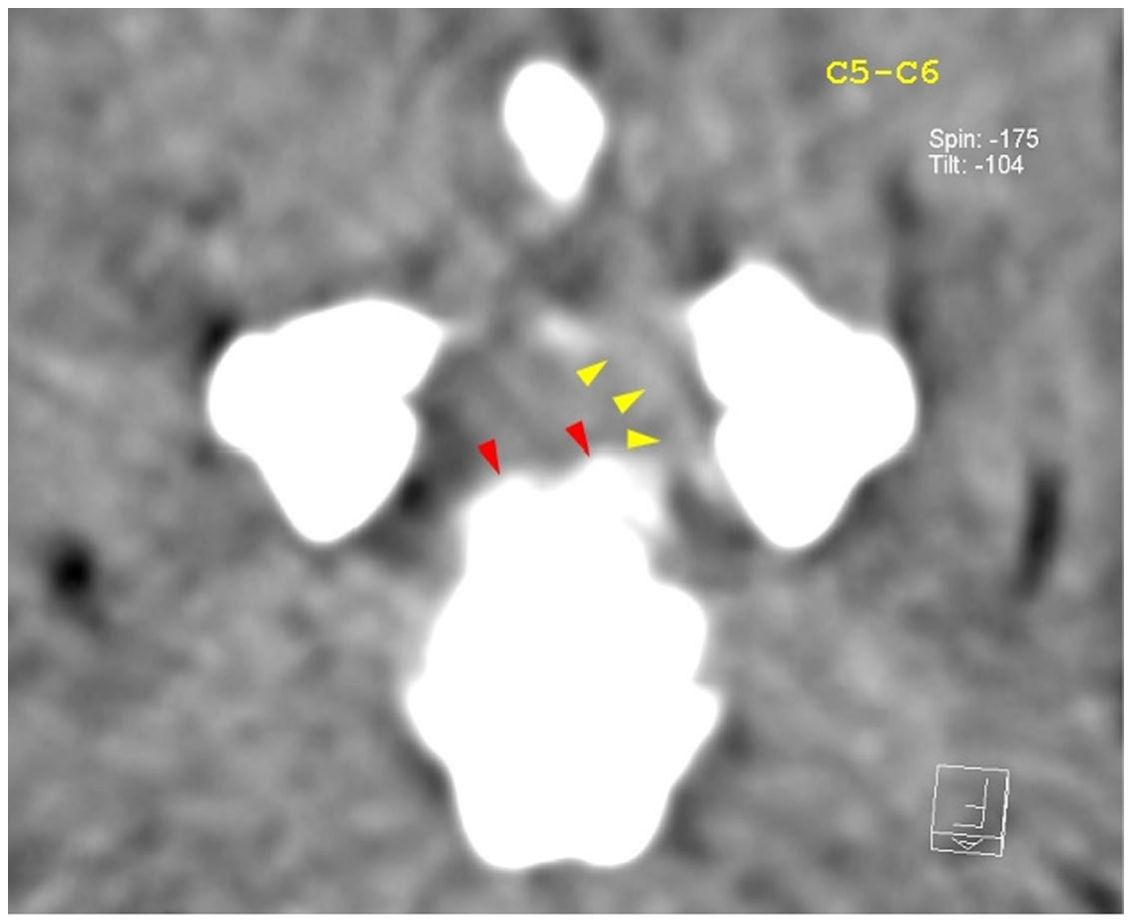
Transverse view at the level of the C5–C6 intervertebral disk space. Red arrowheads highlight the mineralized component of the herniated disk, while the yellow arrowheads highlight mild hyperattenuating disk material with possible hemorrhage.

**Figure 6:**
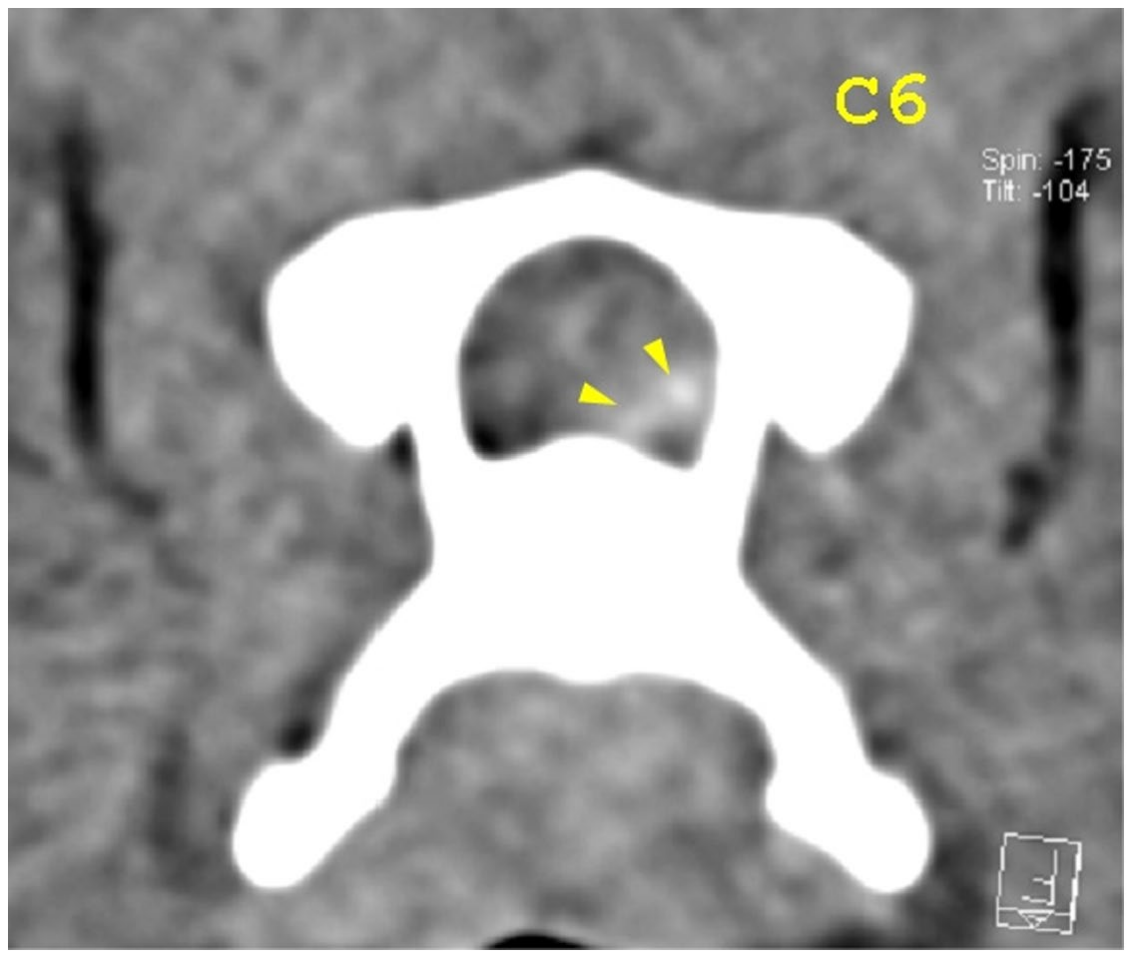
Transverse view at the caudal end of the C6 vertebra. Yellow arrowheads highlight mild hyperattenuating disk material with possible hemorrhage.

**Figure 7:**
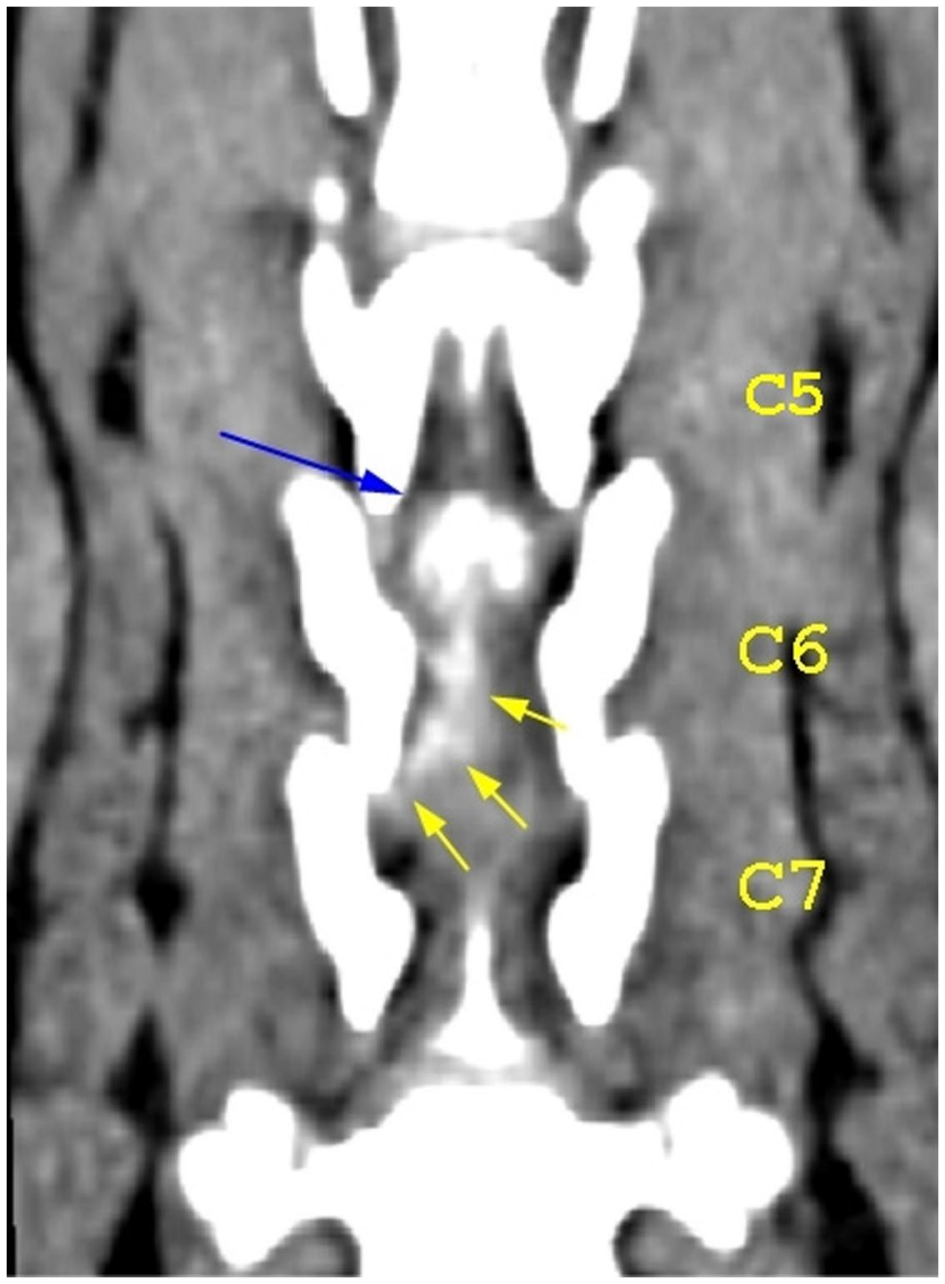
Dorsal view of the caudal cervical segment of the vertebral column. The blue arrow highlights the mineralized component of the herniated disk at the level of the C5–C6 intervertebral disk space; the yellow arrows highlight the caudal extension of the herniated disk material.

The CT scan revealed hyperattenuating heterogeneous material present in the vertebral canal at the level of intervertebral disk space C5–C6, with caudal extension near the C6–C7 intervertebral disk space and minimal cranial extension. The material was located ventral and right lateral to the spinal cord, presented higher HU values (97–185) compared to the spinal cord (12–51), and occupied 45% of the vertebral canal diameter. The C5–C6 intervertebral disk space was markedly narrowed when compared to the adjacent intervertebral spaces (Figures 4–7). These findings were consistent with the Hansen type 1 disk extrusion with moderate spinal cord compression.

Both dogs were tetraplegic with an upper motor neuron deficit in the thoracic limbs; this finding is suggestive of C1–C5 cervical spine pathology. Although both dogs had some degree of lower motor neuron deficit in the pelvic limbs, there was no abnormality detected at the level of the thoracolumbar spinal cord.

The CT findings were discussed with the owners, and the treatment chosen for both of the dogs was to surgically decompress the spinal cord by performing a double adjacent ventral slot. For the first case, a standard ventral slot was performed between the C4–C5 and C5–C6 intervertebral disk spaces, and a fenestration was performed between the C3 and C4 intervertebral disk spaces. For the second case, a standard ventral slot was performed between the C5–C6 and C6–C7 intervertebral disk spaces. Both patients were prepared immediately for the surgical procedure, as described by Platt & da Costa (22).

Fluid therapy was initiated using a balanced crystalloid solution of sodium chloride 0.9% at a rate of 10 mL/kg/h to maintain hydration and electrolyte balance. The premedication consisted of the administration of 0.3 mg/kg/IV of methadone and 0.01 mg/kg/IV of medetomidine, and the general anesthesia was induced with 0.5–1 mg/kg/IV of propofol. Both patients were intubated, and the anesthesia was maintained with isoflurane and oxygen. A dose of 22 mg/kg of cefuroxime was administered approximately 20 min prior to the skin incision, and it was repeated one more time after 90 min, during the surgical procedure. The total duration of the procedure for case 1 was 150 min and for case 2 was 170 min.

Both dogs were placed in dorsal recumbency and prepared for surgery and a single dose of 10 mg/kg/IV of paracetamol and 0.2 mg/kg/IV of meloxicam was administered in the preoperative period. Hemorrhage was controlled using bipolar cautery. A *Standard Ventral Slot* was performed for both patients by using an electric drill and a combination between a 3 mm and a 4 mm burr. The size of the slot was 30% of the vertebral body length and approximately 50% of the vertebral width. For case 1, a prophylactic fenestration was performed between C3 and C4, using a no. 11 surgical blade and a curette, as mineralized material was present *in situ* at that level on the CT scan. The skin was closed in a routine fashion using 3/0 USP polydioxanone, and no attempt to place a drain was made as it was not considered necessary.

Both dogs recovered well from anesthesia, and no complication was noted during the procedure and the recovery time. Ice packing was performed for approximately 10–20 min every 4 h in the first 24 h after the surgery to prevent excessive swelling and seroma formation.

The postoperative treatment included the administration of meloxicam 0.1 mg/kg PO for 5 days, paracetamol 10 mg/kg PO for 2 days, and gabapentin 10–20 mg/kg/8 h PO for 7 days. A second dose of 0.3 mg/kg/IV of methadone was repeated at 4 h after the first administration. Only two doses of methadone were administrated for both patients as the pain score showed no necessity for supplementary analgesics. The modified Glasgow Composite Pain Scale was used for both patients for the time being hospitalized. Both patients were evaluated by the surgeon in the morning, using the composite pain scale, and the analgesic protocol was guided by the total pain score.

Both patients were hospitalized for 5 days postoperatively, and the diet regime during this period was made using resting energy requirement (RER) calculations. Both patients were ambulatory at the time of discharge, and the owners were advised to enforce strict rest for the next 21 days, and only short walks on a leash were permitted during this period. Follow-up examinations were performed 21 days postoperatively, and the modified Glasgow Composite Pain Scale showed no signs of pain at the surgical site or on the hyperflexion or hyperextension of the neck. The neurologic signs were completely restored, and the owners did not report any complications during this period.

Both dogs came for follow-up examinations at 3 months and 6 months after the surgery, and their owners reported normal activity with no signs of neurologic dysfunction.

## Discussion

The ventral slot procedure is one of the most common neurosurgical procedures used to treat cervical intervertebral disk herniation in dogs (18). Many other procedures have been described for cervical decompression, including disk fenestration, dorsal laminectomy, hemilaminectomy, and the slanted ventral slot (23, 24). The standard ventral slot is the most commonly used procedure because the direction of the herniated disk usually occurs dorsomedially and also this technique provides access to the floor of the vertebral canal compared to other techniques (1, 18)..

Several complications have been associated with this procedure, such as hemorrhage from the venous sinus, vertebral subluxation, hypoventilation, and insufficient exposure to the lesions that are lateralized, which can lead to incomplete spinal cord decompression. Although other techniques seem more appropriate for lateralized disk material, other authors have reported good clinical outcomes by using a ventral slot even for lateralized disk material (16, 18, 25, 26).

In one of our cases (case 2—Beagle), it was reported that there was minimal bleeding from the venous sinus that resolved with lavage with sterile saline solution at 37°C in the slot and waiting for 5 min. Apart from this inconvenience, no other complication was noted in any of our cases.

In 2020, Kang et al. (27) described the advantages and limitations of the vertebral window provided by a standard ventral slot when compared with a slanted ventral slot, and the results showed that both techniques have limitations, especially in cases where the disk have migrated caudally. The modified slanted ventral slot provides good access to the herniated disk as long as the disk has not migrated caudally, with obvious limitations reported for C3–C4 and C5–C6 intervertebral disk spaces. Considering the location and the large amount of disks that need to be removed for both of our patients, a standard ventral slot procedure was used for both of them. The author recommends that rigorous presurgical planning based on CT imaging should help in the selection of the most appropriate surgical technique that will allow sufficient disk removal and a good clinical outcome.

In 2020, Guo et al. (28) conducted a retrospective study in which they compared the clinical outcome between two groups of canine patients who suffered from single- or multiple-site cervical disk herniation. A total of 123 dogs underwent a single standard ventral slot procedure and 62 dogs underwent multiple-site standard ventral slot procedures. From the total number of dogs that underwent multiple ventral slot decompression, none of the dogs were large breeds, and only two dogs were included in the medium breed category. The author also reported that two of the dogs from the multiple-site ventral slot category showed the following outcome: one of them died of possible cardiac arrest 3 days postoperatively and the other did not become ambulatory at day 4 postoperatively, and no data were reported after that. Considering that only 2 patients were of medium breed and there is currently no information about a double adjacent ventral slot in a large breed dog, it would have been helpful if the author had reported the category to which the two patients belonged.

Merbl et al. (19) reported in 2017 the outcome after performing a triple adjacent ventral slot in a toy breed (Pomeranian) suffering from severe extradural compression due to mineralized disk extrusions between C3–C4, C4–C5, and C5–C6. The author reports that the size and shape of the slot were modified to prevent any postoperative destabilization of the cervical spine, and a cervical splint was also used in the postoperative period to minimize neck movement. The planning of the slot was performed based on the CT findings in a way that will allow complete removal of the herniated disk material. The length of all the slots was approximately 25% smaller than a standard ventral slot, and the width was smaller in C4–C5 and C5–C6 when compared with C3–C4. We can presume that these changes in the width and the length of the ventral slot could have a significant contribution to the prevention of postoperative complications, such as vertebral subluxation, as mentioned in the literature (25). In this case presentation, the correlation between the CT findings and the intraoperative aspect of the retrieved disk material suggests that the disk extrusion between C3 and C4 was highly incriminating for the clinical signs. Overall, the clinical outcome for this particular case was good, with the author suggesting that although the patient improved significantly postoperatively, minor pelvic limb ataxia and proprioceptive deficits were present (19).

In 2022, Olender et al. (20) reported a good long-term prognosis in four small breed dogs (French bulldogs) suffering from extensive cervical IVDE that were treated with double adjacent ventral slots. In all four cases, the extruded material was located ventrolaterally, and the herniated content also had a hemorrhagic component. However, the author investigates the fact whether the hemorrhagic component of the extruded material does or does not require a surgical decompression or would resolve spontaneously over time. Considering that the origin and the amount of the hemorrhage can vary at that level and that in the absence of an MRI, it is hard to assess the extent of the intradural/intramedullary damage, and also the fact that hemorrhage is associated with high risks of tissue liquefication and increased intramedullary pressure, leading to myelomalacia (29), we recommend that any sort of compression at this level should be managed by surgical decompression.

We used a CT scan to evaluate our patients and found specific lesions that matched their neurolocalization. Although the literature suggests that a non-contrast CT scan is a good option to accurately evaluate canine patients with IVDD, especially if the lesion corresponds with the neurolocalization (30–32), we suggest that magnetic resonance imaging would have been a better option if intradural or intramedullary lesions were present.

An inverted cone technique is recommended as it involves less bone removal, resulting in lower risks of hemorrhage and vertebral subluxation (22, 33). In both of our cases, a standard ventral slot was performed, where the length of the slot was approximately 50% of the total length of the vertebra and its width was approximately 25–27% of the total width of the vertebra. In case 1, a triple ventral slot was considered for C3–C4 due to the presence of mineralized disk material; however, since there was no compression at that level, a prophylactic fenestration was performed instead.

Both our cases showed 100% clinical recovery, with no signs of recurrence during a 2-year follow-up period. We advised the owners to look out for any signs of neck pain that could be related to vertebral subluxation during this period, but no such signs were reported in the follow-up period. Even though both of our patients were 7-year-old, intact, medium-sized dogs, the small number of cases does not allow us to conclude the incidence of double adjacent disk herniation in these specific patients.

To date, data about the treatment of dogs with multiple adjacent cervical disk herniations are limited. Our case presentation shows good clinical outcomes following this procedure in two medium-sized dogs with multiple cervical disk herniations.

## Data availability statement

The raw data supporting the conclusions of this article will be made available by the authors, without undue reservation.

## Ethics statement

Ethical approval was not required for the studies involving animals in accordance with the local legislation and institutional requirements because the reason we did not require ethical approval is that the treatment that we applied was for patients who presented to the small animal hospital with pathologies that required a specific therapeutic surgical treatment that involved a combination of validated surgical procedures previously described. Written informed consent was obtained from the owners for the participation of their animals in this study.

## Author contributions

RC: Conceptualization, Investigation, Methodology, Validation, Writing – original draft, Writing – review & editing. BS: Formal analysis, Investigation, Software, Writing – original draft, Writing – review & editing. CG: Conceptualization, Formal analysis, Writing – original draft, Writing – review & editing. AG: Formal analysis, Writing – original draft. GO: Investigation, Validation, Visualization, Software, Writing – review & editing. IT: Supervision, Validation, Writing – review & editing. RL: Supervision, Validation, Writing – review & editing.
